# Improving the accuracy of cardiac DTI by averaging the complex data

**DOI:** 10.1186/1532-429X-17-S1-O38

**Published:** 2015-02-03

**Authors:** Andrew D Scott, Sonia Nielles-Vallespin, Pedro Ferreira, Laura-Ann McGill, Dudley J Pennell, David Firmin

**Affiliations:** 1 Cardiovascular Biomedical Research Unit, The Royal Brompton Hospital, London, UK; 2 National Heart and Lung Insitute, Imperial College, London, UK; 3 Division of Transmural Research - National Heart Lung and Blood Institute, National Institutes of Health, Bethesda, MD, USA

## Background

When performing cardiac diffusion tensor imaging (cDTI) multiple averages are typically acquired to compensate for the low signal to noise ratio of the individual images. However, the potential for reducing noise in low signal areas is not fully realized when the averaging is performed on the magnitude data[1]. Averaging the complex cDTI data is not straightforward as the diffusion weighting introduces a different, spatially varying phase across each image. In this work we use simulations to demonstrate the benefits available when using complex averaging and then develop an algorithm for performing complex averaging of *in-vivo* cDTI data, which accounts for the induced phase variations.

## Methods

To compare the performance of magnitude and complex averaging in the presence of noise, simulated noisy diffusion weighted images of the left ventricle were calculated with b-values from 50-3000smm^-2^ based on previously published *in-vivo* cDTI data[2]. Magnitude or complex averaging was performed before processing the images with the software we typically use for *in-vivo* cDTI data.

Additionally, *in-vivo* cDTI was performed on a Siemens Skyra in 10 subjects using the STEAM-EPI sequence[3] with 4 b-values, 500-2000smm^-2^, 12 averages, 6 directions, SENSE reconstruction (magnitude and phase images) and 2.8x2.8x8mm^3^ resolution. In order to remove motion-induced phase, each image (Real+Imaginary) was filtered (pyramid mask in k-space, extent: ¼ field-of-view[4]) and the phase of the low-resolution copy was removed from the original images. The images were then processed as for the simulations.

## Results

**Figure 1:**
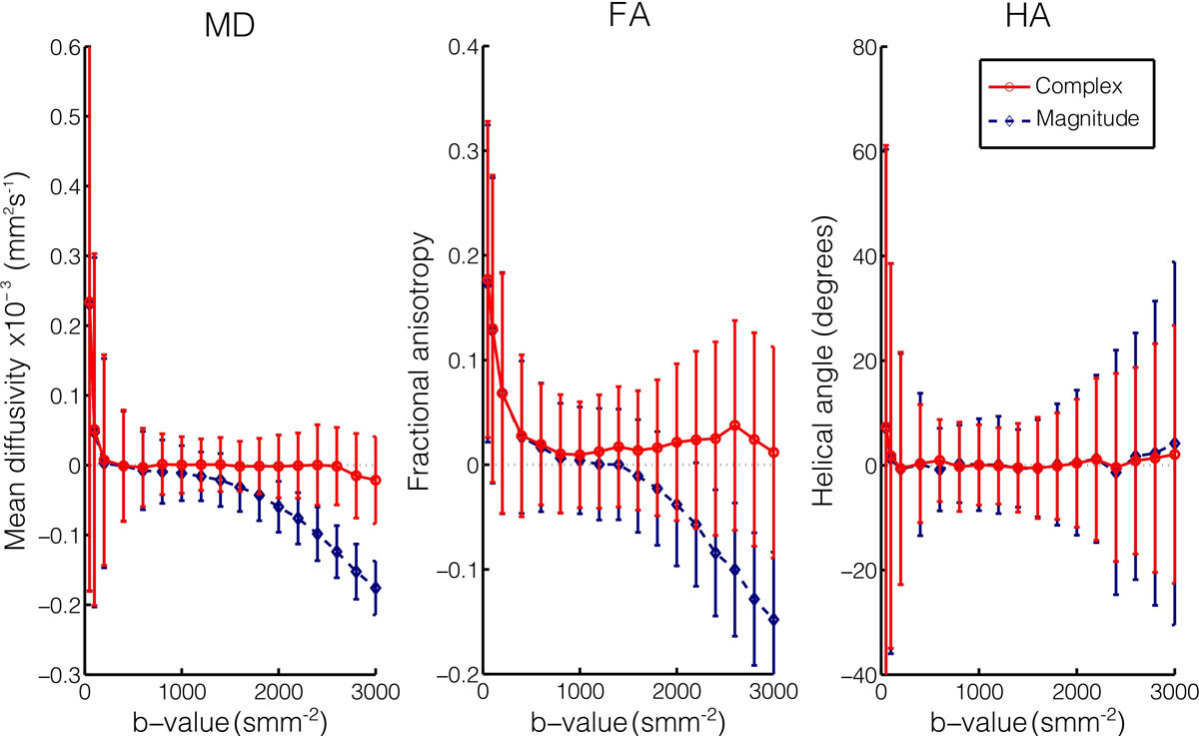
Simulations showing the mean difference ± standard deviations in cDTI derived parameters (mean diffusivity: MD, fractional anisotropy: FA, helical angle: HA) from the ground truth with a range of b-values. The simulated SNR was 17 in the unaveraged reference images and 11 averages were used. The error is calculated as [simulated result] - [ground truth] so a positive bias indicates an over-estimation of the parameter.

Figure 1 shows the results of simulations demonstrating the errors in mean diffusivity (MD), fractional anisotropy (FA) and helical angle (HA) for a range of b-values. At low b-values, no benefit from complex averaging is observed, but at high b-values >1000smm^-2^, the underestimation of MD and FA using magnitude averaging ("squashing the peanut"[5]) can be compensated for by using complex averaging.

**Figure 2:**
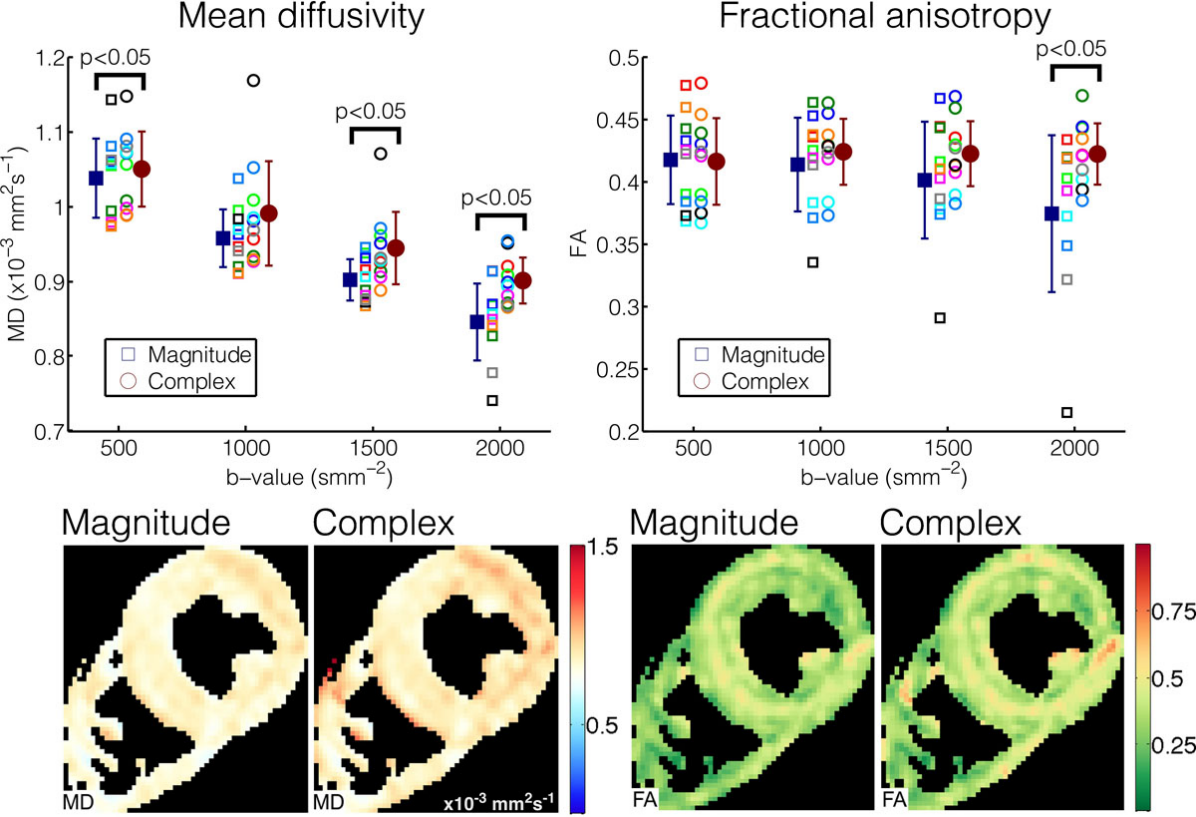
*In-vivo* results showing the mean diffusivity and fractional anisotropy for all 10 subjects (upper plots) at b=500, 1000, 1500 and 2000smm^-2^. Results at each b-value were compared with a paired t-test and significant results are indicated on the plots. Example mean diffusivity and fractional anisotropy maps from one subject are also shown from data acquired with b=2000smm^-2^, highlighting the recovery of lost MD and FA at high b-values when the complex averaging algorithm is used.

Figure 2 compares results obtained *in vivo*, processed with magnitude and complex averaging. As predicted by the simulations, FA and MD are increased when using complex averaging and changes in the parameter maps are evident.

## Conclusions

Large b-values are required to avoid under-estimation of FA and MD in cDTI. We have demonstrated that the phase introduced by diffusion weighting *in vivo* can be corrected for within the reconstruction, allowing us to partially compensate for the noise-floor effects present at high b-values by using complex averaging.

## Funding

This work was performed at the National Institute for Health Research Funded Cardiovascular Biomedical Research Unit at The Royal Brompton Hospital and Imperial College.
